# Supplementary material for: Identification of a Novel Cuproptosis-Related Gene Signature for Prognostic Implication in Head and Neck Squamous Carcinomas
Source: Cancers (Basel). 2022 Aug 18;14(16):3986. doi: 10.3390/cancers14163986 (PMC9406337; doi:10.3390/cancers14163986)
Supplement: Supplementary file 1 [file cancers-14-03986-s001.zip › legned of the Supplementary Figure S1.pdf]

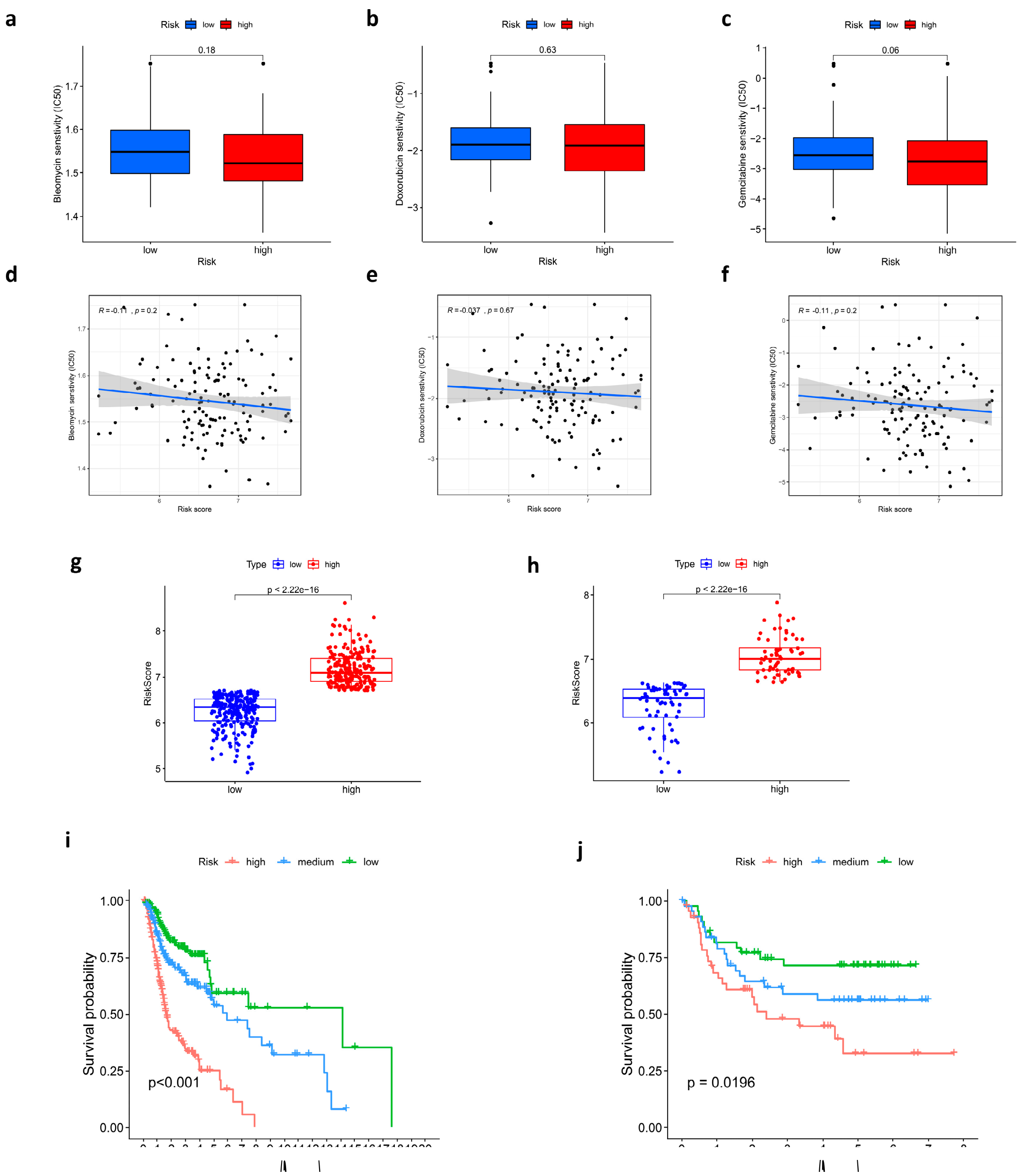

Figure S1. (a-c) Comparison of estimated IC50 value of bleomycin (a), doxorubicin (b), and gemcitabine (c) between low- and high-risk score groups in the GEO cohort. (d-f) The correlation between risk scores and estimated IC50 value of bleomycin (d), doxorubicin (e), and gemcitabine (f) in the GEO cohort. (g) The distribution of the risk score between the two groups in the TCGA cohort. (h) The distribution of the risk score between the two groups in the GEO cohort. (i) OS was compared between low-, medium-, and high-risk score groups in the TCGA cohort. (j) OS was compared between low-, medium-, and high-risk score groups in the GEO cohort.
